# Using three‐dimensional ultrasound in predicting complex gastroschisis: A longitudinal, prospective, multicenter cohort study

**DOI:** 10.1002/pd.5568

**Published:** 2019-10-25

**Authors:** Annelieke Hijkoop, Chiara C.M.M. Lap, Moska Aliasi, Eduard J.H. Mulder, William L.M. Kramer, Hens A.A. Brouwers, Robertine van Baren, Eva Pajkrt, Anton H. van Kaam, Caterina M. Bilardo, Lourens R. Pistorius, Gerard H.A. Visser, René M.H. Wijnen, Dick Tibboel, Gwendolyn T.R. Manten, Titia E. Cohen‐Overbeek

**Affiliations:** ^1^ Department of Pediatric Surgery and Intensive Care Children Erasmus Medical Center—Sophia Children's Hospital, Erasmus University Rotterdam Rotterdam The Netherlands; ^2^ Department of Obstetrics, Division Woman and Baby University Medical Center UtrechtUtrecht University Utrecht The Netherlands; ^3^ Department of Pediatric Surgery University Medical Center Utrecht, Utrecht University Utrecht The Netherlands; ^4^ Department of Neonatology, Division Woman and Baby University Medical Center Utrecht, Utrecht University Utrecht The Netherlands; ^5^ Department of Pediatric Surgery University Medical Center Groningen, University of Groningen Groningen The Netherlands; ^6^ Department of Obstetrics and Gynecology, Amsterdam Reproduction and Development Research Institute Amsterdam University Medical Center, University of Amsterdam Amsterdam The Netherlands; ^7^ Department of Neonatology Emma Children's Hospital, Amsterdam University Medical Center, University of Amsterdam Amsterdam The Netherlands; ^8^ Department of Obstetrics and Gynecology University Medical Center Groningen, University of Groningen Groningen The Netherlands; ^9^ Department of Obstetrics and Gynecology University of Stellenbosch Stellenbosch South Africa; ^10^ Department of Obstetrics and Gynecology, Division of Obstetrics and Prenatal Medicine Erasmus MC—Sophia Children's Hospital, Erasmus University Rotterdam Rotterdam The Netherlands

## Abstract

**Objective:**

To determine whether complex gastroschisis (ie, intestinal atresia, perforation, necrosis, or volvulus) can prenatally be distinguished from simple gastroschisis by fetal stomach volume and stomach‐bladder distance, using three‐dimensional (3D) ultrasound.

**Methods:**

This multicenter prospective cohort study was conducted in the Netherlands between 2010 and 2015. Of seven university medical centers, we included the four centers that performed longitudinal 3D ultrasound measurements at a regular basis. We calculated stomach volumes (n = 223) using Sonography‐based Automated Volume Count. The shortest stomach‐bladder distance (n = 241) was determined using multiplanar visualization of the volume datasets. We used linear mixed modelling to evaluate the effect of gestational age and type of gastroschisis (simple or complex) on fetal stomach volume and stomach‐bladder distance.

**Results:**

We included 79 affected fetuses. Sixty‐six (84%) had been assessed with 3D ultrasound at least once; 64 of these 66 were liveborn, nine (14%) had complex gastroschisis. With advancing gestational age, stomach volume significantly increased, and stomach‐bladder distance decreased (both *P* < .001). The developmental changes did not differ significantly between fetuses with simple and complex gastroschisis, neither for fetal stomach volume (*P* = .85), nor for stomach bladder distance (*P* = .78).

**Conclusion:**

Fetal stomach volume and stomach‐bladder distance, measured during pregnancy using 3D ultrasonography, do not predict complex gastroschisis.

## Abstract 2

What's already known about this topic?

- Infants with complex gastroschisis have a higher risk of morbidity than those with simple gastroschisis.
- Many attempts have been made to prenatally predict complex gastroschisis, using two‐dimensional ultrasound parameters.

What does this study add?

- This longitudinal prospective multicenter study is the first to evaluate the possible benefit of the use of three‐dimensional ultrasound in fetuses with gastroschisis.
- Fetal stomach volume and stomach‐bladder distance, measured during pregnancy using three‐dimensional ultrasound, cannot predict complex gastroschisis.

**What's already known about this topic?**

- Infants with complex gastroschisis have a higher risk of morbidity than those with simple gastroschisis.
- Many attempts have been made to prenatally predict complex gastroschisis, using two‐dimensiona ultrasound parameters.

**What does this study add?**

- This longitudinal prospective multicenter study is the first to evaluate the possible benefit of the use of three‐dimensional ultrasound in fetuses with gastroschisis.
- Fetal stomach volume and stomach‐bladder distance, measured during pregnancy using three‐dimensional ultrasound, cannot predict complex gastroschisis.

## 1 INTRODUCTION

Gastroschisis is an abdominal wall defect that is diagnosed prenatally in over 90% of the cases, usually before 23 weeks' gestation.^1^ In countries that offer routine ultrasound scans at 11 to 14 weeks' gestation, gastroschisis is usually diagnosed in the first trimester.^2^ This allows for early parental counseling and adjustment of obstetric management.

Seventeen percent of all neonates with gastroschisis are diagnosed with additional intestinal defects at birth, ie, intestinal atresia, perforation, necrosis, or volvulus (defined as complex gastroschisis).^3^, ^4^ Infants with complex gastroschisis have a higher risk of morbidity than those with simple gastroschisis; they often experience prolonged time to full enteral feeding (TFEF), more complications, and prolonged length of hospital stay (LOS).^3^, ^4^, ^5^, ^6^

Prenatal detection or prediction of complex gastroschisis would lead to more complete parental counseling. The association between two‐dimensional (2D) prenatal ultrasound findings (eg, bowel dilatation, stomach dilatation, or amniotic fluid index) and complex gastroschisis has been investigated in a number of studies, which showed conflicting results.^7^ Intra‐abdominal bowel dilatation has been associated with intestinal atresia, but its positive predictive value is debatable.^8^ Fetal stomach dilatation has been associated with neonatal death, but not with complex gastroschisis.^8^ However, volume calculation using 2D ultrasound measurements assumes certain geometric characteristics and regular contours of the stomach, which may not be accurate. Three‐dimensional (3D) ultrasound might be more accurate in measuring fetal stomach volume and thus predicting complex gastroschisis, but to date there are no studies to support this hypothesis.

One study used magnetic resonance imaging (MRI) to describe fetal development in case of gastroschisis.^9^ Extensive contact was seen between the stomach and urinary bladder in all but the youngest third trimester fetus who presented with simple gastroschisis at birth. In contrast, those fetuses presenting with intestinal stenosis had not shown any stomach‐bladder contact, as their abdominal cavity was filled with dilated bowel loops.^9^ Therefore, stomach‐bladder distance might be a reflection of intra‐abdominal bowel dilatation (IABD) and may predict complex gastroschisis.

The primary aim of this study was to define whether fetal stomach volume, measured longitudinally using 3D ultrasound, can predict complex gastroschisis. In addition, we aimed to evaluate the value of stomach‐bladder distance in predicting complex gastroschisis in 3D ultrasound volumes.

## 2 METHODS

Between June, 2010 and April, 2015, we performed a prospective, longitudinal, multicenter cohort study at seven university medical centers with a prenatal and a pediatric surgery department in The Netherlands. The centers that performed longitudinal 3D ultrasound measurements on fetuses with gastroschisis at a regular basis (ie, if ≥50% of included fetuses had ≥1 assessment) were included. Fetuses were eligible for inclusion if gastroschisis without any extra‐gastrointestinal anomaly was confirmed by prenatal ultrasound. Neonates who presented with unexpected additional extra‐gastrointestinal anomalies at birth were excluded post‐hoc. This study was approved by the Medical Ethical Review Board of University Medical Center Utrecht. Parents gave written informed consent.

### 2.1 Ultrasound examinations

Advanced ultrasound examinations were planned at 20, 24, 28, 30, 32, 34, 35, and 36 weeks' gestation for longitudinal measurements. 2D ultrasound measures are described elsewhere (ie, fetal biometry, amniotic fluid index, pulsatility indices of the umbilical and superior mesenteric artery, and bowel diameter measurements; manuscript accepted for publication).^10^ 3D volumes of the fetal abdomen were obtained if logistically possible (settings: coronal or sagittal plane; sectional planes with speckle reduction imaging [SRI] and X‐beam activated; quality: high). The volume sample box was adjusted to include the entire fetal abdomen, but as narrow as possible to shorten the acquisition time. The acquisition of the volume was repeated if movement artifacts were detected. All examinations were performed by three to five trained ultrasonographers per center, using a General Electric Voluson 730 or E8 (General Electric Healthcare, London) ultrasound machine, with a 4 to 8‐MHz transabdominal transducer.

**Figure 1:**
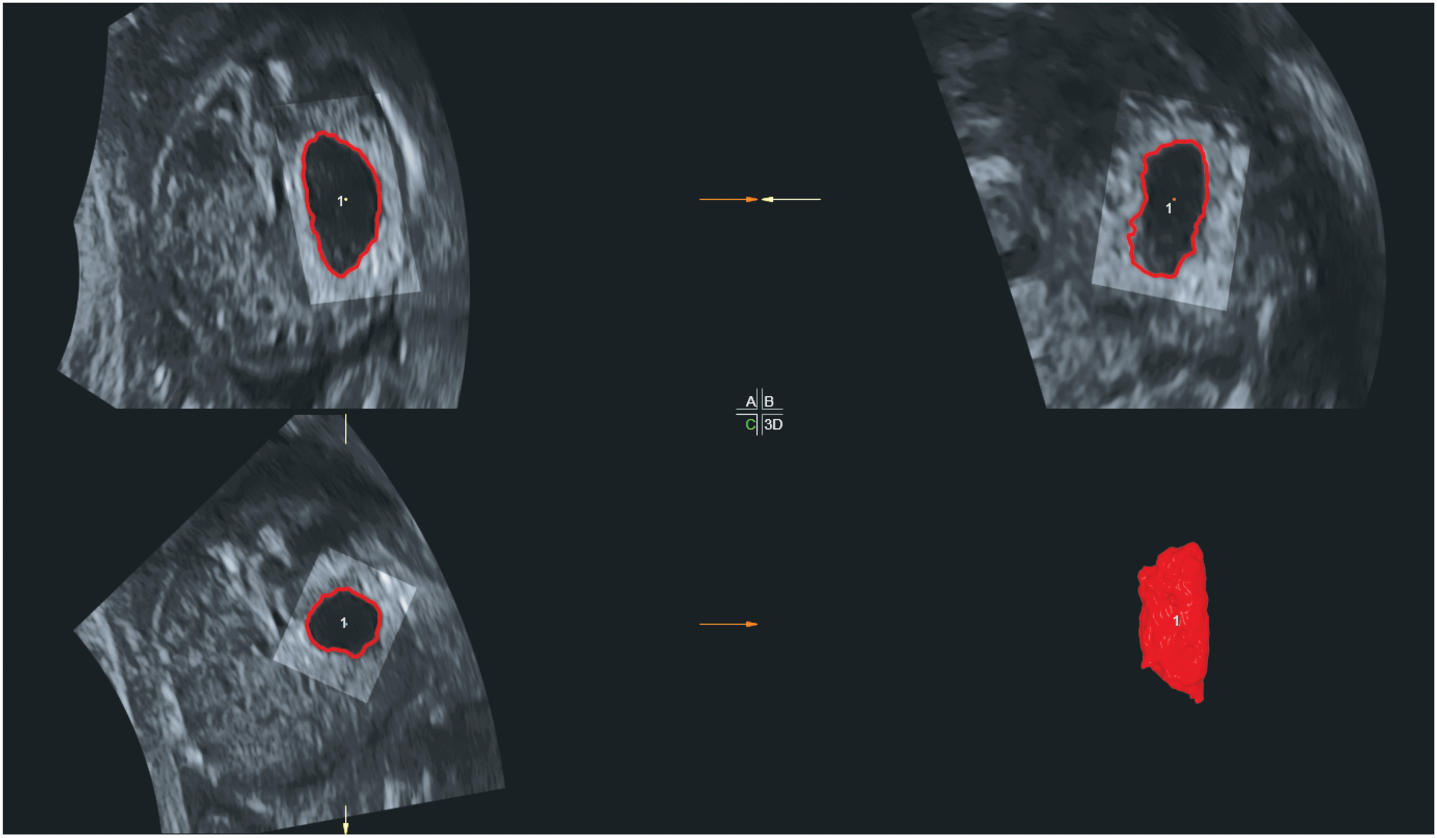
Fetal stomach volume at 21 weeks' gestation, measured using Sonography‐based Automated Volume Count (SonoAVC) [Colour figure can be viewed at http://wileyonlinelibrary.com]

To calculate fetal stomach volumes, we used the Sonography‐based Automated Volume Count (SonoAVC) method.^11^ Each volume was analyzed using 4D View V14 Ext. 4. After uploading the volume dataset, we used multiplanar visualization and positioned the reference point in the center of the stomach in all three planes. We started volume analysis and selected the smallest box possible (Figure 1). After activating SonoAVC general, stomach volumes were calculated by right clicking inside the stomach walls (Figure 1). If necessary, we used the edit mode to cut or merge contours. Volume datasets were excluded if they did not include the stomach, or if insufficient image quality or presence of debris hampered SonoAVC to calculate a volume.

**Figure 2:**
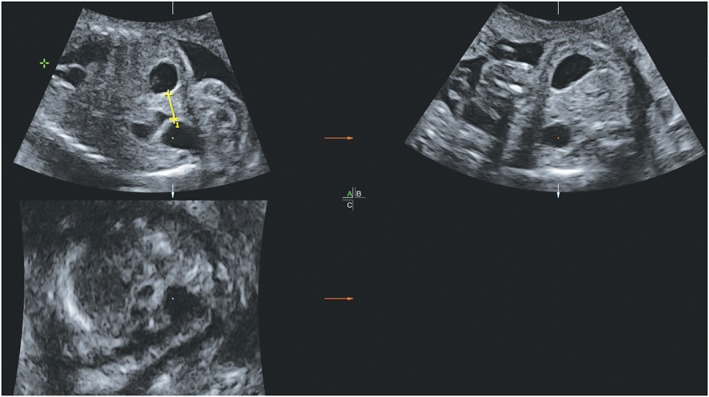
Fetal stomach‐bladder distance at 24 weeks' gestation (yellow markers and line), measured in multiplanar visualization [Colour figure can be viewed at http://wileyonlinelibrary.com]

To measure the shortest stomach‐bladder distance, from outer wall to outer wall, we used the multiplanar visualization of the volume datasets (Figure 2). Volume datasets were excluded from analysis if they did not include the stomach or bladder, or if image quality was insufficient for stomach‐bladder distance calculation. All volumes were analyzed by one investigator (A.H.), who was blinded to the type of gastroschisis.

### 2.2 Variables and definitions

We documented maternal, perinatal, and postnatal characteristics of infants with simple and complex gastroschisis. Complex gastroschisis was defined as gastroschisis complicated by intestinal atresia, volvulus, perforation and/or necrosis at primary evaluation at birth. Neonates were classified as small for gestational age (SGA) if their birth weight was below the 10^th^ percentile according to Dutch reference curves.^12^ If infants needed parenteral nutrition for over 2 years, TFEF was documented as 730 days. Data of deceased infants were excluded from TFEF and LOS analyses.

### 2.3 Statistical analysis

Categorical variables were presented as number (%) and continuous variables as median (interquartile range, IQR). We compared maternal, perinatal, and postnatal characteristics between infants with simple and complex gastroschisis using the chi‐square tests or Fisher's exact tests (in case of expected counts <5) for categorical data, and the Mann‐Whitney U test for continuous data. A two‐sided *P* value of <.05 was considered statistically significant. Statistical analyses were performed using SPSS V.21.0.

### 2.4 Intraobserver and interobserver reliability and agreement

A random subset of 30 stomach volumes was analyzed twice by one investigator (A.H.) to determine intraobserver agreement. The same subset was analyzed by a second independent investigator (M.A.) to determine interobserver agreement. A different subset, also consisting of 30 volumes, was used to determine intraobserver and interobserver agreement of stomach‐bladder distance measurements. We constructed Bland‐Altman plots using the absolute difference between measurements against their mean. The intraobserver and interobserver reliability was estimated by calculating the 95% limits of agreement.^13^ In addition, intraobserver and interobserver agreement scores were assessed by calculating the intraclass correlation coefficient (ICC) with 95% confidence interval (CI).

### 2.5 Longitudinal 3D ultrasound measurements

Non‐normally distributed data were natural log (ln) transformed. If the fetal stomach was adjacent to the bladder (value zero), stomach‐bladder distance was registered as 0.01 cm. We used linear mixed modeling to evaluate the effects of gestational age (GA), type of gastroschisis (simple or complex), and their interaction on the developmental courses of fetal stomach volume and stomach‐bladder distance. Mixed‐effects models allow for intrafetal correlation of repeated measurements, make use of the exact age at measurement, and account for a dissimilar number of measurements on each fetus. Such models also allow for individual variation in growth trajectories, as random effects permit variability in intercept and slope between subjects. We explored linear and quadratic terms of GA that were included as both fixed and random effects. Type of gastroschisis was included as a main effect and also as an interaction with the GA terms. Model estimates are presented as mean and 95% CI.

## 3 RESULTS

**Figure 3:**
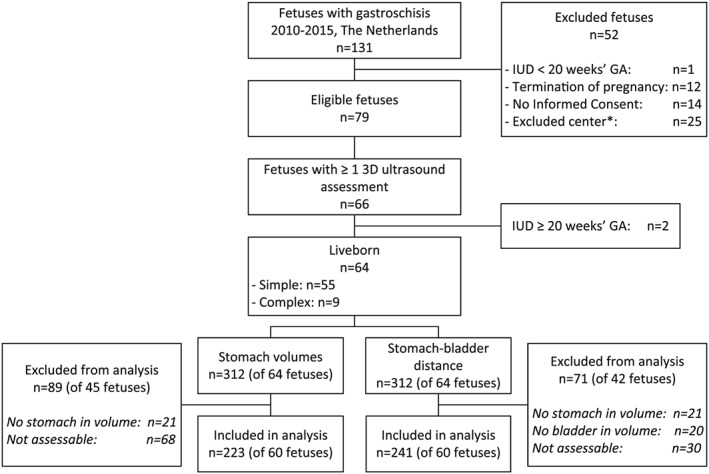
Flow chart of fetuses with gastroschisis included in analyses of stomach volume and stomach‐bladder distance. *Centers were excluded if <50% of included fetuses had ≥1 assessment. IUD: intra‐uterine demise; GA: gestational age

During the study period, 131 fetuses were diagnosed with gastroschisis in The Netherlands. Twenty‐seven (21%) fetuses were excluded: one pregnancy resulted in intra‐uterine demise (IUD) before 20 weeks' gestation, 12 couples opted for termination of the pregnancy, and 14 couples did not want to participate in this study (Figure 3). In addition, three out of seven university medical centers did not perform longitudinal 3D ultrasound measurements on a regular basis; fetuses from these three centers (n = 25) were excluded. No statistically significant differences in maternal, perinatal, or postnatal characteristics were found between infants who were included in our study and those who were excluded, apart from the proportion of neonates delivered by cesarean section which was almost four times higher in the included neonates (*P* = .023, Table S1).

The remaining four centers included 79 fetuses, of which 66 (84%) had been assessed with 3D ultrasound at least once. Two (3%) of these pregnancies resulted in IUD at 28 and 33 weeks' gestation, respectively, and nine of the remaining 64 (14%) live born neonates were diagnosed with complex gastroschisis.

A total of 312 3D ultrasound examinations were performed (Figure 3): 275 in 55 fetuses with simple gastroschisis (mean [range] per fetus: 5 [1‐11]), and 37 in nine fetuses with complex gastroschisis (mean [range] per fetus: 4 [1‐7]). Eighty‐nine stomach volumes of 45 fetuses and 71 stomach‐bladder distances of 42 fetuses were excluded from analysis (eg, due to insufficient quality). In the 89 volume datasets that were excluded from analysis of stomach volume, the proportion of volumes derived from fetuses with complex gastroschisis (20/89, 22%) was significantly higher than that in the total number of volumes available (37/312, 12%) (*P* = .011).

We included a total of 223 stomach volume calculations: 206 of 52 fetuses with simple gastroschisis (mean [range] per fetus: 4 [1‐9]), and 17 of eight fetuses with complex gastroschisis (mean [range] per fetus: 2 [1‐5]). We included a total of 241 stomach‐bladder distances: 216 of 53 fetuses with simple gastroschisis (mean [range] per fetus: 4 [1‐9]), and 25 of seven fetuses with complex gastroschisis (mean [range] per fetus: 4 [1‐8]). Eight fetuses had only one stomach volume calculation available, and for three fetuses only one stomach‐bladder distance could be calculated.

### 3.1 Intraobserver and interobserver reliability and agreement

We found a high degree of intraobserver reliability for both stomach volume (ICC: 0.997, 95% CI: 0.988‐0.999) and stomach‐bladder distance calculations (ICC: 0.931, 95% CI: 0.861‐0.966).

The same was true for interobserver reliability (ICC: 0.981, 95% CI: 0.955‐0.991 for stomach volume, and ICC: 0.962, 95% CI: 0.950‐0.992 for stomach‐bladder distance calculations). Bland‐Altman plots showed good intraobserver and interobserver agreement for both stomach volume and for stomach‐bladder distance calculations (mean intraobserver and interobserver differences with 95% limits of agreement are shown in Figure S1).

### 3.2 Maternal, perinatal, and postnatal characteristics

Neonates with complex gastroschisis were born 1.5 weeks earlier than those with simple gastroschisis, but this difference did not reach statistical significance. Infants with complex gastroschisis were over three times more likely to develop cholestatic jaundice than those with simple gastroschisis (Table 1). In addition, wound infections were over six times more prevalent in the complex gastroschisis group. Median TFEF was more than 6 months in infants with complex gastroschisis, compared with less than 1 month in infants with simple gastroschisis. Median LOS was 4 months in infants with complex gastroschisis, and 1 month in those with simple gastroschisis. One infant with complex gastroschisis died of sepsis at 8 months of age.

**Table 1:** Maternal, perinatal, and postnatal characteristics of included live born infants (n = 64, from four centers) with simple or complex gastroschisis

	n	Simple Gastroschisis n = 55 (86%)	n	Complex Gastroschisis a n = 9 (14%)	*P* Value
Number of 3D assessments	55	5 (4‐7)	9	4 (2‐6)	0.30
Maternal characteristics					
Age (years)	54	25 (22‐30)	9	24 (22‐29)	0.54
Primigravid	55	31 (56%)	9	4 (44%)	0.72
Smoking	49	17 (35%)	8	3 (38%)	1.00
Recreational drug use b	50	6 (12%)	8	2 (25%)	0.30
Perinatal characteristics					
Gestational age at birth (weeks)	55	36.9 (35.7‐37.4)	9	35.4 (33.5‐37.0)	0.06
Spontaneous onset of delivery	55	13 (24%)	9	4 (44%)	0.23
Cesarean section	55	16 (29%)	9	4 (44%)	0.44
Birth weight (grams)	55	2565 (2230‐2775)	9	2220 (1840‐2800)	0.23
Birth weight < p10	55	8 (15%)	9	3 (33%)	0.18
Male gender	55	25 (45%)	9	5 (56%)	0.72
Apgar at 5 min < 7	54	3 (6%)	9	1 (11%)	0.47
Postnatal characteristics					
Primary closure	55	34 (62%)	9	5 (56%)	0.73
Complications c	55	28 (51%)	9	8 (89%)	0.07
‐ Necrotizing enterocolitis		0 (0%)		1 (11%)	0.14
‐ Cholestatic jaundice		13 (24%)		7 (78%)	0.003
‐ Line sepsis		18 (33%)		5 (56%)	0.26
‐ Wound infection		3 (5%)		3 (33%)	0.03
Mortality	55	0 (0%)	9	1 (11%)	0.14
Time to full enteral feeding (days)	54	28 (17‐42)	8	201 (98‐386)	0.001
Length of hospital stay (days) ^d^	55	34 (25‐63)	8	122 (71‐180)	0.001

Data presented as median (interquartile range) or n (%).

a Intestinal atresia (n = 6), intestinal atresia + perforation (n = 1), intestinal atresia + necrosis (n = 1), intestinal atresia + necrosis + volvulus (n = 1).

b Simple gastroschisis: cocaine (n = 4), marihuana (n = 2); complex gastroschisis: cocaine (n = 1), marihuana (n = 1).

c Percentages do not necessarily add up to 100, as one infant can have multiple problems. One infant with complex gastroschisis died of sepsis at 8 months of age.

One infant with simple gastroschisis and one with complex gastroschisis were transferred to another hospital with an unknown discharge date to home; in these infants, length of hospital stay was documented as time to transfer.

### 3.3 Developmental course of stomach volume and stomach‐bladder distance

**Figure 4:**
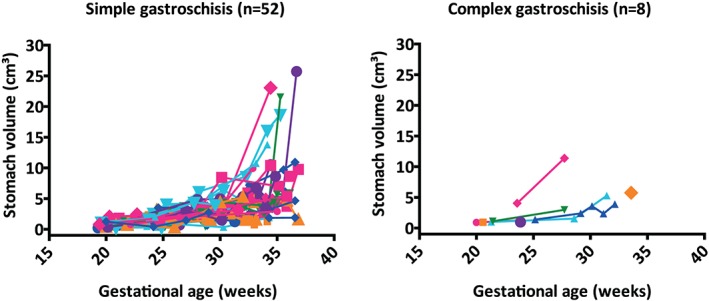
Stomach volumes in fetuses with simple or complex gastroschisis during gestational age. Different colors and symbols represent different fetuses.Location of intestinal atresia in complex gastroschisis (n = 8): *jejunal* (pink rhombus, green triangle); *jejunal + colonic* (pink circle); *ileal* (light blue triangle, dark blue triangle, orange rhombus); *unclear* (orange square, purple circle) [Colour figure can be viewed at http://wileyonlinelibrary.com]

**Figure 5:**
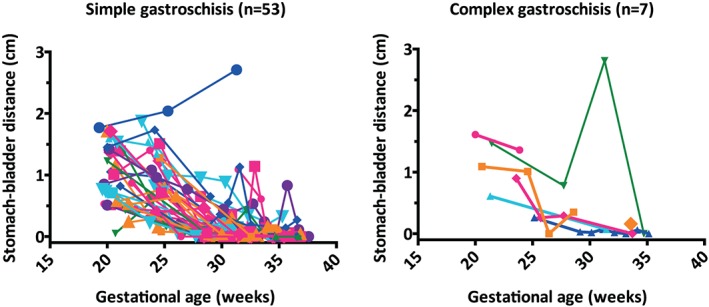
Stomach‐bladder distances in fetuses with simple or complex gastroschisis during gestational age. Different colors and symbols represent different fetuses.Location of intestinal atresia in complex gastroschisis (n = 7): *jejunal* (pink rhombus, green triangle); *jejunal + colonic* (pink circle); *ileal* (light blue triangle, dark blue triangle, orange rhombus); *unclear* (orange square) [Colour figure can be viewed at http://wileyonlinelibrary.com]

Linear mixed modeling showed no significant contribution of a GA‐squared term; a linear model fitted the ln‐transformed data best. Fetal stomach volume did not differ significantly between fetuses with simple and those with complex gastroschisis at 20 weeks' gestation (Figure 4, Table 2; *P* = .397), nor did stomach‐bladder distance (Figure 5, Table 2; *P* = .345). With advancing GA, stomach volume significantly increased, and stomach‐bladder distance decreased (both *P* < .001). The course of these changes did not differ significantly between simple and complex gastroschisis (Table 2).

**Table 2:** Estimates with 95% confidence intervals of linear mixed modeling for stomach volume and stomach‐bladder distance (natural log transformed)

Variable	Estimate (Mean)	95% Confidence Interval	*P* Value
Stomach volume (ln)			
Intercept	−0.31	−0.49 to −0.13	0.001
Type of gastroschisis (complex versus simple)	0.25	−0.33 to 0.83	0.40
Gestational age (centered at 20 weeks)	0.13	0.11 to 0.15	<0.001
Gestational age by type of gastroschisis	0.01	−0.07 to 0.08	0.85
Stomach bladder distance (ln)			
Intercept	0.06	−0.27 to 0.39	0.71
Type of gastroschisis (complex versus simple)	0.48	−0.53 to 1.48	0.35
Gestational age (centered at 20 weeks)	−0.26	−0.30 to −0.22	<0.001
Gestational age by type of gastroschisis	−0.02	−0.15 to 0.11	0.78

The infant who died of sepsis at 8 months of age had shown normal stomach volume at 24 weeks' gestation. Stomach‐bladder distance was not assessable; no 3D ultrasound measurements were available between 24 and 33 weeks' gestation for this infant. The infant was born at 33 weeks' gestation with an appropriate birth weight for GA.

For the two pregnancies resulting in IUD (beyond 20 weeks' gestation), we found fetal stomach volume and stomach‐bladder distance comparable to those shown in Figures 4 and 5, respectively. Neither had any other structural malformations at autopsy. The autopsy report of one fetus mentioned intestinal malrotation; the report of the other fetus stated signs of placental inflammation without specifically addressing intestinal malrotation.

## 4 DISCUSSION

This longitudinal prospective multicenter study is the first to evaluate the possible benefit of the use of 3D ultrasound in fetuses with gastroschisis. Stomach volume and stomach‐bladder distance during pregnancy did not differ between simple and complex gastroschisis. Therefore, we were unable to predict complex gastroschisis using these prenatal variables.

Many attempts have been made to prenatally predict complex gastroschisis.^8^ Fetal stomach dilatation has been found to be associated with the postnatal need for bowel resection,^14^ but a recent meta‐analysis showed no significant association between stomach dilatation and complex gastroschisis.^8^ However, stomach dilatation in these fetuses was always evaluated retrospectively, using 2D ultrasound.^8^ In addition, the cut‐off values used in these studies were either not mentioned^14^, ^15^ or were derived from healthy fetuses more than 30 years ago.^8^, ^16^, ^17^ In our group of more than 100 fetuses that were evaluated with 2D ultrasound, we found that both intraabdominal and extraabdominal bowel diameters were of limited value in the prediction of complex gastroschisis.^10^ Although both parameters were increased in those with complex gastroschisis, the large fluctuations over time and the overlap with simple cases made it difficult to identify complex gastroschisis prenatally. The best predictor appeared to be intraabdominal bowel diameters ≥p97.7 measured at least three times during gestation, but the positive predictive value was low (ie, 50%). Gastric size was not assessed in the 2D ultrasound part of the study.

As 3D ultrasound has been proposed to be superior to 2D ultrasound in evaluating fetal stomach volume,^18^ we hypothesized that this method would be more accurate in predicting complex gastroschisis. However, fetuses with complex gastroschisis showed stomach volumes comparable with those measured in simple gastroschisis fetuses.

Previous studies have reported an association between fetal stomach dilatation and death in the neonatal^8^ or perinatal^14^ period. In our study, the two cases ending in IUD had stomach volumes that were comparable to those who were live born. No previous study has evaluated the association between fetal stomach‐bladder distance and complex gastroschisis. *Brugger and Prayer*, however, did report extensive stomach‐bladder contact on MRI in fetuses who presented with simple gastroschisis at birth.^9^ This was in contrast to the three fetuses with complex gastroschisis included in their study, who had shown absence of stomach‐bladder contact in the third trimester due to IABD.^9^ As IABD has previously been associated with complex gastroschisis,^8^ we hypothesized that a greater stomach‐bladder distance—as a reflection of IABD—could also be predictive of complex gastroschisis. Rather than measuring the largest bowel loop, stomach‐bladder distance would reflect IABD in general. However, both in simple and in complex gastroschisis, we observed great variations in stomach‐bladder distance, probably due to alternate filling and emptying of these organs. As no differences were observed between the two types of gastroschisis, we conclude that stomach‐bladder distance is not helpful in predicting complex gastroschisis.

### 4.1 Strengths and limitations

The major strength of our study is its prospective, longitudinal study design, with a large number of measurements per fetus. Investigators were blinded to outcome during ultrasonography and during calculations of stomach volume and stomach‐bladder distance. As we used 3D instead of 2D ultrasonography, we did not depend on certain geometric characteristics or regular contours of the stomach to calculate stomach volume, and we were able to reliably calculate the shortest stomach‐bladder distance.

Several limitations need to be addressed. First, we excluded three centers because of low compliance of performing 3D ultrasound measurements. However, we found no significant differences in characteristics between cases of included centers and cases of excluded centers, apart from the number of cesarean sections. Therefore, we expect that selection bias can be considered minimal. Second, the small sample of fetuses with complex gastroschisis decreased the power of our tests. Since these fetuses showed comparable stomach volume and stomach‐bladder distance to those with simple gastroschisis, we think this has not affected our conclusion. A third limitation is the substantial number of missing data for fetuses from included centers. Especially in the complex gastroschisis group, a relatively high number of volume datasets had to be excluded from stomach volume analyses, because no stomach was seen intraabdominally or because volume calculations were not assessable. We speculate that fetuses with complex gastroschisis may have an increased incidence of stomach evisceration, or increased presence of debris inside the stomach, which hampered SonoAVC to calculate stomach volumes. Nonetheless, all fetuses with complex gastroschisis included in our analysis showed comparable stomach volumes to those with simple gastroschisis. Even if the excluded volume datasets would have shown strongly deviating values, it would still be very difficult to predict complex gastroschisis using stomach volume. Last, we chose to focus on 3D ultrasound measures only. Future research may investigate whether combining 3D with 2D ultrasound measures leads to improved prediction of complex gastroschisis.

## 5 CONCLUSION

We conclude that fetal stomach volume and stomach‐bladder distance, measured during pregnancy using 3D ultrasonography, cannot predict complex gastroschisis.

## CONFLICT OF INTEREST

none declared.

## FUNDING

none declared.

## Supporting information


**Figure S1.** Bland‐Altman plots showing intraobserver and interobserver agreement of stomach volume and stomach‐bladder distance measurements.

**Table S1.** Maternal, perinatal, and postnatal characteristics of fetuses with gastroschisis from included and excluded centers.

## Data Availability

data are available upon reasonable request.
