# Supplementary material for: Using three‐dimensional ultrasound in predicting complex gastroschisis: A longitudinal, prospective, multicenter cohort study
Source: Prenat Diagn. 2019 Oct 25;39(13):1204–12. doi: 10.1002/pd.5568 (PMC6972561; doi:10.1002/pd.5568)
Supplement: Supplementary file 2 — Table S1. Maternal, perinatal, and postnatal characteristics of fetuses with gastroschisis from included and excluded centers. [file PD-39-1204-s002.docx]

**SUPPLEMENTAL TABLES**

**Supplemental Table 1.** Maternal, perinatal and postnatal characteristics of fetuses with gastroschisis from included and excluded centers.

|  | n | Included centers (n=4)  79 fetuses |  | Excluded centers (n=3)  25 fetuses | p-value |
| --- | --- | --- | --- | --- | --- |
| ≥ 1 3D ultrasound assessment | 79 | 66 (84%) | 25 | 7 (28%) | <0.001 |
| Maternal characteristics |  |  |  |  |  |
| Age (years) | 78 | 25 (22 – 31) | 25 | 26 (23 – 31) | 0.64 |
| Primigravid | 79 | 40 (51%) | 25 | 14 (56%) | 0.64 |
| Smoking | 68 | 24 (35%) | 25 | 10 (40%) | 0.68 |
| Recreational drug use | 69 | 13 (19%) | 24 | 2 (8%) | 0.34 |
| Perinatal characteristics |  |  |  |  |  |
| Livebirth | 79 | 75 (95%) | 25 | 25 (100%) | 0.57 |
| Gestational age at birth (weeks) | 75 | 36.7 (35.3 – 37.3) | 25 | 36.7 (35.6 – 37.1) | 0.93 |
| Spontaneous onset of delivery | 75 | 23 (31%) | 25 | 9 (36%) | 0.62 |
| Cesarean section | 75 | 23 (31%) | 25 | 2 (8%) | 0.02 |
| Birth weight (grams) | 75 | 2490 (2175 – 2775) | 25 | 2395 (2165 – 2770) | 0.82 |
| Birth weight < p10 | 75 | 13 (17%) | 25 | 3 (12%) | 0.75 |
| Male gender | 75 | 38 (51%) | 25 | 15 (60%) | 0.42 |
| Apgar at 5 min < 7 | 74 | 4 (5%) | 25 | 1 (4%) | 1.00 |
| Postnatal characteristics |  |  |  |  |  |
| Complex gastroschisis | 75 | 13 (17%) | 25 | 6 (24%) | 0.56 |
| Primary closure | 75 | 44 (59%) | 24 | 19 (79%) | 0.07 |
| Complications ^†^ | 75 | 45 (60%) | 25 | 15 (60%) | 1.00 |
| - Necrotizing enterocolitis |  | 1 (1%) |  | 1 (4%) | 0.44 |
| - Cholestatic jaundice |  | 26 (35%) |  | 10 (40%) | 0.63 |
| - Line sepsis |  | 27 (36%) |  | 11 (44%) | 0.48 |
| - Wound infection |  | 10 (13%) |  | 3 (12%) | 1.00 |
| Mortality | 75 | 3 (4%) | 25 | 0 (0%) | 0.57 |
| Time to full enteral feeding (days) | 71 | 29 (19 – 70) | 23 | 36 (23 – 49) | 0.79 |
| Length of hospital stay (days) ^‡^ | 72 | 43 (26 – 81) | 24 | 44 (27 – 80) | 0.81 |

Data presented as median (interquartile range) or n (%).

^†^ Percentages do not necessarily add up to 100, as one infant can have multiple problems.

^‡^ Three infants in the included group and one in the excluded group were transferred to another hospital with an unknown discharge date to home; in these infants, length of hospital stay was documented as time to transfer.
